# Supplementary material for: Identification of photoperiod-induced specific miRNAs in the adrenal glands of Sunite sheep (Ovis aries)
Source: Front Vet Sci. 2022 Jul 22;9:888207. doi: 10.3389/fvets.2022.888207 (PMC9354845; doi:10.3389/fvets.2022.888207)
Supplement: Supplementary file 1 [file Data_Sheet_1.ZIP › Supplementary file/miRDeep2 file.zip!/miRDeep2 file/Know_Structure/LP42A2/oar-mir-199a.pdf]

miRBase precursor : oar-mir-199a
Total read count : 151423
oar-miR-199a-3p read count : 98217
remaining reads : 53206

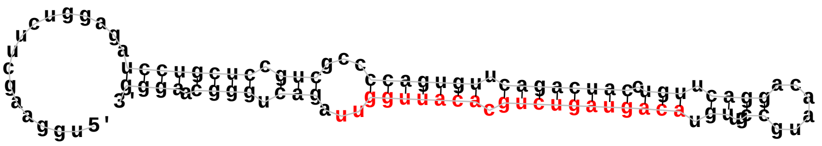

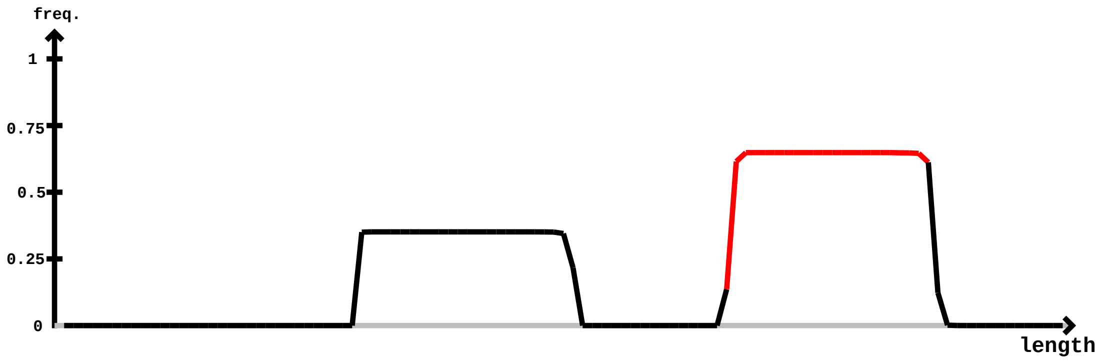


```
                                                                                    oar-miR-199a-3p
5'- uggaagcuucuggagauccugcuccgucgccccaguguucagacuaccuguucaggacaaugccguuguacaguagucugcacauugguuagacugggcaaggg   -3'    exp
    .................(((((((.(((...(((((((.((((((((.(((.((((......))..)).))))))))))).)))))))...))).)))).))).   reads    mm    sample
    .............................ccccaguguucagacuaccuguu....................................................   6        0     seq
    .............................ccccaguguucagacuaccuguuc...................................................   1        0     seq
    ..............................cccaguguucagacuacc........................................................   4        0     seq
    ..............................cccaguguucagacuaccu.......................................................   36       0     seq
    ..............................cccaguguucagacuaccug......................................................   70       0     seq
    ..............................cccaguguucagacuaccugu.....................................................   834      0     seq
    ..............................cccaguguucagacuaccuguu....................................................   19400    0     seq
    ..............................cccaguguucagacuaccuguuc...................................................   32688    0     seq
    ...............................ccaguguucagacuaccugu.....................................................   4        0     seq
    ...............................ccaguguucagacuaccuguu....................................................   46       0     seq
    ...............................ccaguguucagacuaccuguuc...................................................   104      0     seq
    ................................caguguucagacuaccuguu....................................................   7        0     seq
    ................................caguguucagacuaccuguuc...................................................   3        0     seq
    .................................aguguucagacuaccuguuc...................................................   1        0     seq
    ..................................guguucagacuaccuguC....................................................   1        1     seq
    ...................................uguucagacuaccuguu....................................................   1        0     seq
    ....................................................................uacaguagucugcacauu..................   50       0     seq
    ....................................................................uacaguagucugcacauug.................   10       0     seq
    ....................................................................uacaguagucugcacauugg................   118      0     seq
    ....................................................................uacaguagucugcacauuggu...............   2396     0     seq
    ....................................................................uacaguagucugcacauugguu..............   16786    0     seq
    ....................................................................uacaguagucugcacauugguua.............   1299     0     seq
    ....................................................................uacaguagucugcacauugguuag............   6        0     seq
    ....................................................................uacaguagucugcacauugguuaga..........   3        0     seq
    .....................................................................acaguagucugcacauu..................   90       0     seq
    .....................................................................acaCuagucugcacauu..................   1        1     seq
    .....................................................................acaguagucugcacauug.................   36       0     seq
    .....................................................................acaguagucugcacauugg................   127      0     seq
    .....................................................................acaguagucugcacauuggu...............   2492     0     seq
    .....................................................................acaguagucugcacauugguu..............   53635    0     seq
    .....................................................................acaguagucugcacauugguua.............   15921    0     seq
    .....................................................................acaguagucugcacauugguuag............   168      0     seq
    .....................................................................acaguagucugcacauugguuagG...........   1        1     seq
    .....................................................................acaguagucugcacauugguuaga..........   13       0     seq
    ......................................................................caguagucugcacauugg................   12       0     seq
```

oar-miR-199a-3p

```
uggaagcuucuggagauccugcuccgucgccccaguguucagacuaccuguucaggacaaugccguuguacaguagucugcacauugguuagacugggcaaggg
......................................................................caguagucugcacauuggu...............   199    0    seq
......................................................................caguagucugcacauugguu..............   3622   0    seq
......................................................................caguagucugcacauugguua.............   1172   0    seq
......................................................................caguagucugcacauugguuag............   22     0    seq
......................................................................caguagucugcacauugguuaga...........   8      0    seq
......................................................................caguagucugcacauugguuagG...........   1      1    seq
.......................................................................aguagucugcacauugguu..............   18     0    seq
.......................................................................aguagucugcacauugguua.............   11     0    seq
```
